# Effect of Preoperative Hormone Replacement Therapy on Postmenopausal Women Undergoing Rotator Cuff Repair

**DOI:** 10.7759/cureus.110455

**Published:** 2026-06-08

**Authors:** Anh Le, Andrea H Johnson, Jane C Brennan, Matthew A Peterman, James York, Benjamin M Petre, Justin Turcotte, Daniel E Redziniak

**Affiliations:** 1 Orthopedic Research, Anne Arundel Medical Center, Annapolis, USA; 2 Orthopedics, Anne Arundel Medical Center, Annapolis, USA; 3 Orthopedic Surgery, Anne Arundel Medical Center, Annapolis, USA; 4 Orthopedic and Surgical Research, Anne Arundel Medical Center, Annapolis, USA

**Keywords:** estrogen replacement therapy, postmenopausal women, postoperative complications, postoperative outcomes, rotator cuff repair

## Abstract

Background

The effect of exogenous estrogen, as hormone replacement therapy (HRT), on surgical outcomes following rotator cuff repair (RCR) is unclear. This study evaluated the effect of preoperative HRT on postmenopausal women undergoing RCR. We hypothesize that preoperative HRT use is associated with higher rates of 90-day complications and worse two-year outcomes after RCR.

Methodology

A retrospective review of 22,992 female patients undergoing RCR in the PearlDiver database was performed. Patients on HRT within one year preoperatively were matched 1:3 with those not on HRT. Overall, 5,748 on HRT and 17,244 not on HRT were included. Univariate and multivariate analyses were performed to compare outcomes between groups.

Results

After propensity score matching, HRT patients had higher rates of alcohol disorders (p = 0.008), rheumatologic disorders (p = 0.001), and osteoporosis (p = 0.008), as well as an overall increased comorbidity burden, as shown by an increased Charlson Comorbidity Index score (p < 0.001). At two years postoperatively, the HRT group had increased rates of prolonged opioid use (3,958 (68.9%) vs. 10,527 (61.0%); p < 0.001) and total shoulder arthroplasty (123 (2.1%) vs. 296 (1.7%); p = 0.043). After controlling for the remaining differences between groups, the HRT group was still 40% more likely to have prolonged opioid use at two years post RCR (odds ratio: 1.40, 95% confidence interval: 1.31 to 1.49; p < 0.001). When comparing the type of HRT use, those who used oral HRT had increased rates of prolonged opioid use (3,090 (70.1%) vs. 868 (64.7%); p < 0.001) and total shoulder arthroplasty (104 (2.4%) vs. 19 (1.4%); p = 0.048) at two years postoperatively compared to those who used transdermal HRT.

Conclusions

Based on this study, preoperative HRT appears safe regarding repair integrity, although surgeons could consider risk stratifying patients based on administration route and implementing multimodal pain management strategies for patients on oral therapies.

## Introduction

Menopause, characterized by a significant decline in estrogen levels, marks a critical transition in a woman’s life that has substantial impacts on musculoskeletal health. Estrogen is essential for maintaining skeletal integrity by modulating bone formation and resorption. In its absence, bone turnover increases, leading to reduced bone mineral density and an increased risk of osteoporosis and associated fragility fractures [1]. Hormone replacement therapy (HRT), often prescribed for menopausal symptoms, preserves bone mineral density and reduces osteoporotic fracture risk [2]. HRT was widely prescribed before the early 2000s when the Women’s Health Initiative trials noted significantly increased risks of cardiovascular events, breast cancer, and dementia [3,4]. Subsequently, black box warnings were placed on HRT approved for menopausal symptoms and prescribing of these medications decreased precipitously [5]. More recent studies have provided additional nuance to the risks and benefits of HRT, and prescriptions of these medications for menopausal symptoms are again increasing [3,5]. In addition, most of the black box warnings for estrogen-based HRT products have recently been removed, which will likely contribute to an increasing number of prescriptions for these products [4].

The effect of HRT on outcomes following major orthopedic procedures has been a subject of growing interest, yielding a complex and sometimes contradictory body of evidence. Preoperative HRT is associated with a lower risk of periprosthetic femoral fracture after total hip arthroplasty, yet it is also associated with a higher long-term risk of revision and mechanical complications after total shoulder arthroplasty (TSA) [6,7]. This complexity is further highlighted by findings that estrogen deficiency is associated with a higher incidence of rotator cuff repair (RCR), while preclinical animal models suggest estrogen supplementation improves the histologic structure of the healing tendon enthesis and suppresses inflammation [8,9]. These contradictory findings suggest a complex relationship between systemic hormone levels, local joint environments, and surgical outcomes.

While the impact of HRT has been investigated in the context of large joint arthroplasty, there is a notable gap in the literature concerning its effects on soft tissue healing and outcomes in common procedures such as RCR. The biological environment of the tendon-bone interface is critical for healing, and hormonal fluctuations may affect repair integrity and function [9]. Therefore, the purpose of this study was to investigate the impact of preoperative HRT on postoperative outcomes in postmenopausal women undergoing RCR. Specifically, we aimed to evaluate whether HRT use was associated with increased rates of 90-day complications, two-year outcomes, and two-year cost of care following RCR. Additionally, we assessed whether cessation of HRT use within six months before surgery or the route of HRT administration affected outcomes. Based on prior studies of HRT in other orthopedic surgical populations, we hypothesized that preoperative HRT use is associated with higher rates of 90-day complications and worse two-year outcomes after RCR.

## Materials and methods

This study was deemed exempt by the institutional review board as a retrospective review of a de-identified database.

Data source

The PearlDiver (PearlDiver Inc., Colorado Springs, Colorado, USA; www.pearldiverinc.com) Mariner 170 dataset was retrospectively analyzed. The database contains claims records from over 170 million patients across all payers, including commercial, Medicare, Medicaid, and self-pay. Data is searchable by Predefined Cohorts, International Classification of Diseases, Ninth Revision (ICD-9), International Classification of Diseases, Tenth Revision (ICD-10), and Current Procedural Terminology (CPT) codes.

Study population

All patients included in this study underwent arthroscopic or open RCR and were female and at least 55 years old. To be included in this study, patients were required to be active in the database for at least one year before RCR and two years postoperatively. Patients with a history of any of the following comorbidities were excluded from this study: acute myocardial infarction (MI), pulmonary embolism (PE), deep vein thrombosis (DVT), cerebrovascular disease, breast cancer, cervical cancer, ovarian cancer, uterine cancer, or severe liver disease. Patients were divided into groups by whether they filled an HRT prescription within the year before RCR or not. All ICD-9/10 and CPT code definitions of inclusion/exclusion criteria, independent variables, and outcomes can be found in the Appendix for review.

Independent variables

Demographics and comorbidities (based on ICD-9/10 codes) of interest were age, obesity, tobacco use, alcohol disorders, Charlson Comorbidity Index (CCI) score, diabetes, thyroid disorders, depression/anxiety, rheumatologic disorders, primary hypertension, cardiac disorders, osteoporosis, and preoperative steroid injection within the six months before RCR.

Outcome measures

The following outcomes were assessed at 90 days postoperatively: infection and readmission. The following outcomes were assessed at two years postoperatively: TSA, revision RCR, frozen shoulder, stroke, PE, DVT, acute MI, and total cost. Opioid use was considered prolonged use if patients were continuing to fill prescriptions at two years postoperatively.

Statistical analysis

Patients who were not prescribed HRT were propensity score matched 3:1 on all independent variables to patients who were prescribed HRT. After propensity score matching, univariate analyses (chi-square and independent samples t-tests) were performed to compare demographics, comorbidities, and outcomes between groups. Multivariate linear and logistic regression were used to assess the association between HRT use and outcomes, controlling for comorbidities that were significantly different after propensity score matching. Patients prescribed HRT were further grouped by the time of their last HRT use: within six months before RCR and between six to twelve months before RCR. Univariate analyses were performed to compare outcomes between patients whose last HRT use was within six months before surgery and those whose last HRT use was between six and twelve months before surgery. Additionally, patients prescribed HRT were further divided based on the type of HRT used, i.e., oral or transdermal, and univariate analyses were performed to compare outcomes between groups. All statistical analyses were performed within the PearlDiver platform using R (RStudio PBC, Boston, MA). Statistical significance was assessed at p-values <0.05.

## Results

After propensity score matching, 5,748 patients filled an HRT prescription within the year before RCR, and 17,244 patients did not. There were no differences in age, rates of obesity, tobacco use, diabetes, thyroid disorders, depression/anxiety, rheumatologic disorders, primary hypertension, cardiac disorders, and preoperative steroid injections between groups. HRT patients had higher rates of alcohol disorders (97 (1.7%) vs. 209 (1.2%); p = 0.008), rheumatologic disorders (442 (7.7%) vs. 1,112 (6.4%); p = 0.001), and osteoporosis (773 (13.4%) vs. 2,086 (12.1%); p = 0.008). Additionally, those who had HRT had an overall increased comorbidity burden, as shown by an increased CCI score (1.1 ± 1.4 vs. 1.0 ± 1.3, p < 0.001) (Table 1).

**Table 1:** Demographics and comorbidities after propensity score matching. P-values <0.05 are in bold; all data presented as n (%) or mean ± SD. HRT = hormone replacement therapy; CCI = Charlson Comorbidity Index

Demographics and comorbidities	No HRT use (n = 17,244)	HRT use (n = 5,748)	P-value
Age, years	64.3 ± 6.1	64.3 ± 6.1	0.925
Obesity	3,457 (20.0)	1,185 (20.6)	0.363
Tobacco use	2,475 (14.4)	856 (14.9)	0.325
Alcohol disorders	209 (1.2)	97 (1.7)	0.008
CCI	1.0 ± 1.3	1.1 ± 1.4	<0.001
Diabetes	3,350 (19.4)	1,173 (20.4)	0.110
Thyroid disorders	5,588 (32.4)	1,882 (32.7)	0.650
Depression/Anxiety	6,659 (38.6)	2,279 (39.6)	0.169
Rheumatologic disorders	1,112 (6.4)	442 (7.7)	0.001
Primary hypertension	9,859 (57.2)	3,276 (57.0)	0.823
Cardiac disorders	4,165 (24.2)	1,407 (24.5)	0.631
Osteoporosis	2,086 (12.1)	773 (13.4)	0.008
Preoperative steroid injection	2,145 (12.4)	770 (13.4)	0.062

Postoperatively, there were no differences in 90-day readmission or infection between groups. At two years postoperatively, there were no differences in rates of revision RCR, frozen shoulder, acute MI, PE, DVT, and stroke. However, the HRT group had increased rates of prolonged opioid use (3,958 (68.9%) vs. 10,527 (61.0%); p < 0.001), and TSA (123 (2.1%) vs. 296 (1.7%); p = 0.043), as well as increased total cost (17,860 ± 44,650 vs. 15,920 ± 21,614; p < 0.001) at two years (Table 2).

**Table 2:** Ninety-day and two-year outcomes. P-values <0.05 are in bold; all data presented as n (%) or mean ± SD. HRT = hormone replacement therapy; USD = United States dollars

Outcome	No HRT use (n = 17,244)	HRT use (n = 5,748)	P-value
90 days			
Infection	25 (0.1)	10 (0.2)	0.770
Readmission	134 (0.8)	44 (0.8)	1
2 years			
Opioid use	10,527 (61.0)	3,958 (68.9)	<0.001
Total shoulder arthroplasty	296 (1.7)	123 (2.1)	0.043
Revision rotator cuff repair	935 (5.4)	325 (5.7)	0.525
Frozen shoulder	535 (3.1)	188 (3.3)	0.556
Stroke	133 (0.8)	32 (0.6)	0.114
Pulmonary embolism	51 (0.3)	25 (0.4)	0.145
Deep vein thrombosis	127 (0.7)	46 (0.8)	0.692
Acute myocardial infarction	105 (0.6)	37 (0.6)	0.846
Cost (USD)	15,920 ± 21,614	17,860 ± 44,650	<0.001

After controlling for the remaining differences in independent variables between groups, the HRT group was still 40% more likely to have prolonged opioid use at two years after RCR (odds ratio: 1.40, 95% confidence interval: 1.31 to 1.49; p < 0.001). However, HRT use was no longer significantly associated with any other outcomes after matching (Table 3).

**Table 3:** Ninety-day and two-year outcomes: multivariate analysis. Controlling for alcohol disorders, CCI score, rheumatologic disease, and osteoporosis. P-values <0.05 are in bold. HRT = hormone replacement therapy; OR = odds ratio; CI = confidence interval; USD = United States dollars

Outcome	HRT use OR/β	95% CI	P-value
90 days			
Infection	1.06	0.48 to 2.15	0.885
Readmission	0.93	0.65 to 1.30	0.684
2 years			
Opioid use	1.40	1.31 to 1.49	<0.001
Total shoulder arthroplasty	1.21	0.98 to 1.50	0.076
Revision rotator cuff repair	1.04	0.91 to 1.18	0.558
Frozen shoulder	1.04	0.88 to 1.23	0.659
Stroke	0.68	0.45 to 0.99	0.051
Pulmonary embolism	1.49	0.91 to 2.39	0.101
Deep vein thrombosis	1.07	0.75 to 1.49	0.706
Acute myocardial infarction	0.99	0.67 to 1.43	0.958
Cost (USD)	0.0002	-0.0001 to 0.0006	0.258

When comparing the time of last HRT use, there were no differences in outcomes between those who stopped HRT within six months and those who stopped HRT within six to twelve months before RCR (Table 4).

**Table 4:** Ninety-day and two-year outcomes by last HRT use. P-values <0.05 are in bold; all data presented as n (%) or mean ± SD. HRT = hormone replacement therapy; USD = United States dollars

Outcome	Last HRT use, 6 months (n = 2,751)	Last HRT use, 6 to 12 months (n = 2,997)	P-value
90 days			
Infection	3 (0.1)	7 (0.2)	0.415
Readmission	23 (0.8)	21 (0.7)	0.662
2 years			
Opioid use	1,896 (68.9)	2,062 (68.8)	0.946
Total shoulder arthroplasty	48 (1.7)	75 (2.5)	0.059
Revision rotator cuff repair	165 (6.0)	160 (5.3)	0.306
Frozen shoulder	82 (3.0)	106 (3.5)	0.267
Stroke	12 (0.4)	20 (0.7)	0.318
Pulmonary embolism	13 (0.5)	12 (0.4)	0.830
Deep vein thrombosis	20 (0.7)	26 (0.9)	0.653
Acute myocardial infarction	16 (0.6)	21 (0.7)	0.690
Cost (USD)	18,225 ± 59,718	17,525 ± 23,464	0.553

However, when comparing the type of HRT use, those who used oral HRT had increased rates of prolonged opioid use (3,090 (70.1%) vs. 868 (64.7%); p < 0.001) and TSA (104 (2.4%) vs. 19 (1.4%); p = 0.048) at two years postoperatively compared to those who used transdermal HRT (Table 5).

**Table 5:** Ninety-day and two-year outcomes by type of HRT use. P-values <0.05 are in bold; all data presented as n (%) or mean ± SD. HRT = hormone replacement therapy; USD = United States dollars

Outcome	Oral HRT (n = 4,407)	Transdermal HRT (n = 1,341)	P-value
90 days			
Infection	8 (0.2)	3 (0.2)	1
Readmission	36 (0.8)	8 (0.6)	0.528
2 years			
Opioid use	3,090 (70.1)	868 (64.7)	<0.001
Total shoulder arthroplasty	104 (2.4)	19 (1.4)	0.048
Revision rotator cuff repair	256 (5.8)	69 (5.1)	0.393
Frozen shoulder	143 (3.2)	45 (3.4)	0.911
Stroke	27 (0.6)	5 (0.4)	0.410
Pulmonary embolism	18 (0.4)	7 (0.5)	0.752
Deep vein thrombosis	39 (0.9)	7 (0.5)	0.258
Acute myocardial infarction	32 (0.7)	5 (0.4)	0.222
Cost (USD)	17,786 ± 21,202	19,898 ± 82,757	0.125

## Discussion

In this large, propensity-matched cohort of postmenopausal women undergoing RCR, preoperative HRT was not associated with an increased rate of revision surgery, venous thromboembolism (VTE), or other common 90-day postoperative complications, contrary to our initial hypothesis. The principal finding from this analysis was that preoperative HRT use was significantly associated with a 40% increased odds of prolonged opioid use two years after surgery. Further subgroup analysis revealed that this association was primarily driven by patients taking oral HRT, who also demonstrated a higher rate of subsequent conversion to TSA (104 (2.4%) vs 19 (1.4%)) compared to those using transdermal formulations.

The observation that HRT did not influence the rate of revision rotator cuff surgery is notable, particularly when viewed within the context of the existing orthopedic literature. Prior studies have associated hormone therapy with a higher risk of aseptic loosening and revision after total knee and hip arthroplasty, and an increased risk of revision and mechanical complications after TSA [7,10]. Additionally, deficiency of estrogen in female patients has been shown to be associated with increased risk of requiring an RCR [8]. Conversely, other work has shown HRT to be associated with a reduced risk of periprosthetic femoral fracture after total hip arthroplasty, suggesting a bone-protective effect [6]. The neutral effect on revision rates in the present study suggests that the influence of HRT may be highly specific to the underlying tissue biology of the procedure, bone versus soft tissue. While preclinical models indicate that estrogen can improve the histologic features of tendon healing, this potential biologic advantage does not appear to translate into a clinically significant reduction in revision rates in this large population [9]. It is plausible that the multifactorial nature of rotator cuff healing failure, which involves mechanical factors such as tear size and patient factors such as compliance, may overwhelm any subtle biological benefits conferred by HRT.

A significant and unexpected finding was the strong association between preoperative HRT and prolonged postoperative opioid use. This association persisted after controlling for baseline differences in comorbidities, including osteoporosis and rheumatologic conditions, which might predispose patients to chronic pain. The underlying mechanism for this observation is likely complex and multifactorial. While some basic science studies suggest estrogen can modulate central pain perception pathways, a direct link to prolonged postoperative opioid consumption following an orthopedic procedure is not well established and represents a novel clinical finding [11,12]. This association may also be influenced by unmeasured confounders; for example, patients prescribed HRT may have a higher prevalence of baseline chronic pain syndromes or different healthcare-seeking behaviors not fully captured within a claims database. The subgroup analysis provided further insight, revealing that the increased opioid use was driven by patients on oral HRT. This distinction is critical, as oral and transdermal HRT have markedly different systemic effects due to the first-pass metabolism of oral estrogens in the liver [13]. This metabolic route is known to have a greater impact on inflammatory mediators and coagulation factors, which could plausibly influence the postoperative inflammatory response and heighten the pain experience [13]. The parallel finding that the oral HRT group also had a higher rate of conversion to TSA may be clinically related; prolonged, intractable pain and sustained opioid use following a failed RCR are common drivers that lead to a salvage arthroplasty [14].

The lack of an increased risk of VTE across the study aligns with other recent large database studies in orthopedics, suggesting that with modern formulations and appropriate patient selection, perioperative HRT may not carry the significant thrombotic risk that was previously feared [15]. On the contrary, current studies indicate that early initiation of HRT, within 10 years of the onset of perimenopause, may provide health benefits, including reducing the risk of fatal cardiovascular events [4,16,17]. Similarly, a study by Yeh et al. found that patients aged 50-60 years on oral HRT had no increased risk of VTE when compared to patients not on HRT [18]. Other studies have shown that the route of administration of HRT, oral vs. transdermal formulations, does have an impact on VTE risk, with a recent systematic review by Hicks et al. finding no increased risk of VTE in patients using transdermal formulations, but patients on oral formulations, particularly combined estrogen/progestogen, having a higher risk of VTE [19]. There are likely several patient factors that need to be accounted for when assessing risk of VTE and cardiovascular events in patients using HRT for menopausal symptoms, and additional investigation is needed to fully assess this in patients undergoing orthopedic procedures [20].

This study has several limitations inherent to its retrospective design. The reliance on an administrative claims database with diagnostic and procedural coding is susceptible to inaccuracies and prevents us from establishing causation. Furthermore, we were unable to control for key clinical and radiographic variables known to affect rotator cuff healing, such as tear size, tissue quality, surgical technique, or rehabilitation protocols. Additional details regarding the HRT regimen, including dosage, specific formulation, and duration of use, were also not available, which may obscure more nuanced relationships. The definition of prolonged opioid use based on prescription fills is an imperfect proxy for actual consumption. Despite these limitations, the study’s large, propensity-matched cohort provides a valuable real-world perspective. Future prospective studies are necessary to validate these findings and to better understand the mechanisms linking oral HRT to prolonged opioid consumption after rotator cuff surgery.

## Conclusions

In postmenopausal women undergoing RCR, preoperative HRT does not appear to increase the risk of revision surgery, VTE, or 90-day complications. However, HRT use was significantly associated with a 40% increased odds of prolonged opioid use at two years. Subgroup analysis indicated this risk is regimen-dependent, with patients utilizing oral formulations demonstrating higher rates of prolonged opioid use and conversion to TSA compared to those using transdermal formulations. These findings suggest that while HRT is safe regarding repair integrity, surgeons should risk stratify patients based on administration route and implement aggressive multimodal pain management strategies for those on oral therapies.

## Appendices

**Table 6:** Data definitions. ICD = International Classification of Diseases; CPT = Current Procedural Terminology

Group/Measure	ICD/CPT code definitions
Study cohorts	
Rotator cuff repair (RCR)	CPT-29827, CPT-23410, CPT-23412
Hormone Replacement therapy (HRT)	Oral HRT: DRUG-CENESTIN, DRUG-DELESTROGEN, DRUG-ENJUVIA, DRUG-ESTRACE, DRUG-FEMTRACE, DRUG-MENEST, DRUG-OGEN, DRUG-PREMARIN, DRUG-ACTIVELLA, DRUG-ANGELIQ, DRUG-FEMHRT, DRUG-PREFEST, DRUG-PREMPRO, DRUG-DUAVEE Transdermal HRT: DRUG-ALORA, DRUG-CLIMARA, DRUG-CLIMARA_PRO, DRUG-ESTRADERM, DRUG-MENOSTAR, DRUG-MINIVELLE, DRUG-VIVELLE, DRUG-VIVELLE-DOT, DRUG-COMBIPATCH, DRUG-DIVIGEL, DRUG-ELESTRIN, DRUG-ESTRASORB, DRUG-ESTRING, DRUG-EVAMIST, DRUG-FEMRING, DRUG-VAGIFEM
Exclusion criteria	
Acute myocardial infarction	ICD-10-D-I2101, ICD-10-D-I2102, ICD-10-D-I2109, ICD-10-D-I2111, ICD-10-D-I2119, ICD-10-D-I2121, ICD-10-D-I2129, ICD-10-D-I213, ICD-10-D-I214, ICD-10-D-I219, ICD-10-D-I21A1, ICD-10-D-I21A9, ICD-10-D-I220, ICD-10-D-I221, ICD-10-D-I222, ICD-10-D-I228, ICD-10-D-I229, ICD-9-D-41001, ICD-9-D-41011, ICD-9-D-41021, ICD-9-D-41031, ICD-9-D-41051, ICD-9-D-41071, ICD-9-D-41081, ICD-9-D-41091
Cerebrovascular disease	ICD-10-D-G43601, ICD-10-D-G43609, ICD-10-D-G43611, ICD-10-D-G43619, ICD-10-D-I6000, ICD-10-D-I6001, ICD-10-D-I6002, ICD-10-D-I6010, ICD-10-D-I6011, ICD-10-D-I6012, ICD-10-D-I602, ICD-10-D-I6020, ICD-10-D-I6021, ICD-10-D-I6022, ICD-10-D-I6030, ICD-10-D-I6031, ICD-10-D-I6032, ICD-10-D-I604, ICD-10-D-I6050, ICD-10-D-I6051, ICD-10-D-I6052, ICD-10-D-I606 ,ICD-10-D-I607, ICD-10-D-I608, ICD-10-D-I609, ICD-10-D-I610, ICD-10-D-I611, ICD-10-D-I612, ICD-10-D-I613, ICD-10-D-I614, ICD-10-D-I615, ICD-10-D-I616, ICD-10-D-I618, ICD-10-D-I619, ICD-10-D-I6200, ICD-10-D-I6201, ICD-10-D-I6202, ICD-10-D-I6203, ICD-10-D-I621, ICD-10-D-I629, ICD-10-D-I6300, ICD-10-D-I63011, ICD-10-D-I63012, ICD-10-D-I63013, ICD-10-D-I63019, ICD-10-D-I6302, ICD-10-D-I63031, ICD-10-D-I63032, ICD-10-D-I63033, ICD-10-D-I63039, ICD-10-D-I6309, ICD-10-D-I6310, ICD-10-D-I63111, ICD-10-D-I63112, ICD-10-D-I63113, ICD-10-D-I63119, ICD-10-D-I6312, ICD-10-D-I63131, ICD-10-D-I63132, ICD-10-D-I63133, ICD-10-D-I63139, ICD-10-D-I6319, ICD-10-D-I6320, ICD-10-D-I63211, ICD-10-D-I63212, ICD-10-D-I63213, ICD-10-D-I63219, ICD-10-D-I6322, ICD-10-D-I63231, ICD-10-D-I63232, ICD-10-D-I63233, ICD-10-D-I63239, ICD-10-D-I6329, ICD-10-D-I6330, ICD-10-D-I63311, ICD-10-D-I63312, ICD-10-D-I63313, ICD-10-D-I63319, ICD-10-D-I63321, ICD-10-D-I63322, ICD-10-D-I63323, ICD-10-D-I63329, ICD-10-D-I63331, ICD-10-D-I63332, ICD-10-D-I63333, ICD-10-D-I63339, ICD-10-D-I63341, ICD-10-D-I63342, ICD-10-D-I63343, ICD-10-D-I63349, ICD-10-D-I6339, ICD-10-D-I6340, ICD-10-D-I63411, ICD-10-D-I63412, ICD-10-D-I63413, ICD-10-D-I63419, ICD-10-D-I63421, ICD-10-D-I63422, ICD-10-D-I63423, ICD-10-D-I63429, ICD-10-D-I63431, ICD-10-D-I63432, ICD-10-D-I63433, ICD-10-D-I63439, ICD-10-D-I63441, ICD-10-D-I63442, ICD-10-D-I63443, ICD-10-D-I63449, ICD-10-D-I6349, ICD-10-D-I6350, ICD-10-D-I63511, ICD-10-D-I63512, ICD-10-D-I63513, ICD-10-D-I63519, ICD-10-D-I63521, ICD-10-D-I63522, ICD-10-D-I63523, ICD-10-D-I63529, ICD-10-D-I63531, ICD-10-D-I63532, ICD-10-D-I63533, ICD-10-D-I63539, ICD-10-D-I63541, ICD-10-D-I63542, ICD-10-D-I63543, ICD-10-D-I63549, ICD-10-D-I6359, ICD-10-D-I636, ICD-10-D-I638, ICD-10-D-I639, ICD-10-D-I6601, ICD-10-D-I6602, ICD-10-D-I6603, ICD-10-D-I6609, ICD-10-D-I6611, ICD-10-D-I6612, ICD-10-D-I6613, ICD-10-D-I6619, ICD-10-D-I6621, ICD-10-D-I6622, ICD-10-D-I6623, ICD-10-D-I6629, ICD-10-D-I663, ICD-10-D-I668, ICD-10-D-I669, ICD-10-D-R29700, ICD-10-D-R29701, ICD-10-D-R29702, ICD-10-D-R29703, ICD-10-D-R29704, ICD-10-D-R29705, ICD-10-D-R29706, ICD-10-D-R29707, ICD-10-D-R29708, ICD-10-D-R29709, ICD-10-D-R29710, ICD-10-D-R29711, ICD-10-D-R29712, ICD-10-D-R29713, ICD-10-D-R29714, ICD-10-D-R29715, ICD-10-D-R29716, ICD-10-D-R29717, ICD-10-D-R29718, ICD-10-D-R29719, ICD-10-D-R29720, ICD-10-D-R29721, ICD-10-D-R29722, ICD-10-D-R29723, ICD-10-D-R29724, ICD-10-D-R29725, ICD-10-D-R29726, ICD-10-D-R29727, ICD-10-D-R29728, ICD-10-D-R29729, ICD-10-D-R29730, ICD-10-D-R29731, ICD-10-D-R29732, ICD-10-D-R29733, ICD-10-D-R29734, ICD-10-D-R29735, ICD-10-D-R29736, ICD-10-D-R29737, ICD-10-D-R29738, ICD-10-D-R29739, ICD-10-D-R29740, ICD-10-D-R29741, ICD-10-D-R29742, ICD-9-D-34660, ICD-9-D-34661, ICD-9-D-34662, ICD-9-D-34663, ICD-9-D-430, ICD-9-D-431, ICD-9-D-4320, ICD-9-D-4321, ICD-9-D-4329, ICD-9-D-43301, ICD-9-D-43311, ICD-9-D-43321, ICD-9-D-43331, ICD-9-D-43381, ICD-9-D-43391, ICD-9-D-43400, ICD-9-D-43401, ICD-9-D-43410, ICD-9-D-43411, ICD-9-D-43490, ICD-9-D-43491
Cervical cancer	ICD-10-D-C530, ICD-10-D-C531, ICD-10-D-C538, ICD-10-D-C539, ICD-10-D-D060, ICD-10-D-D061, ICD-10-D-D067, ICD-10-D-D069, ICD-10-D-R87610, ICD-10-D-R87611, ICD-10-D-R87612, ICD-10-D-R87613, ICD-10-D-R87614, ICD-10-D-Z8541, ICD-10-D-Z86001, ICD-9-D-1801, ICD-9-D-1808, ICD-9-D-1809, ICD-9-D-2331, ICD-9-D-79501, ICD-9-D-79502, ICD-9-D-79503, ICD-9-D-79504, ICD-9-D-79506, ICD-9-D-V1041
Breast cancer	ICD-10-D-C50011, ICD-10-D-C50012, ICD-10-D-C50019, ICD-10-D-C50021, ICD-10-D-C50022, ICD-10-D-C50029, ICD-10-D-C50111, ICD-10-D-C50112, ICD-10-D-C50119, ICD-10-D-C50121, ICD-10-D-C50122, ICD-10-D-C50129, ICD-10-D-C50211, ICD-10-D-C50212, ICD-10-D-C50219, ICD-10-D-C50221, ICD-10-D-C50222, ICD-10-D-C50229, ICD-10-D-C50311, ICD-10-D-C50312, ICD-10-D-C50319, ICD-10-D-C50321, ICD-10-D-C50322, ICD-10-D-C50329, ICD-10-D-C50411, ICD-10-D-C50412, ICD-10-D-C50419, ICD-10-D-C50421, ICD-10-D-C50422, ICD-10-D-C50429, ICD-10-D-C50511, ICD-10-D-C50512, ICD-10-D-C50519, ICD-10-D-C50521, ICD-10-D-C50522, ICD-10-D-C50529, ICD-10-D-C50611, ICD-10-D-C50612, ICD-10-D-C50619, ICD-10-D-C50621, ICD-10-D-C50622, ICD-10-D-C50629, ICD-10-D-C50811, ICD-10-D-C50812, ICD-10-D-C50819, ICD-10-D-C50821, ICD-10-D-C50822, ICD-10-D-C50829, ICD-10-D-C50911, ICD-10-D-C50912, ICD-10-D-C50919, ICD-10-D-C50921, ICD-10-D-C50922, ICD-10-D-C50929, ICD-10-D-D0500, ICD-10-D-D0501, ICD-10-D-D0502, ICD-10-D-D0510, ICD-10-D-D0511, ICD-10-D-D0512, ICD-10-D-D0580, ICD-10-D-D0581, ICD-10-D-D0582, ICD-10-D-D0590, ICD-10-D-D0591, ICD-10-D-D0592, ICD-10-D-Z853, ICD-10-D-Z86000, ICD-9-D-1741, ICD-9-D-1742, ICD-9-D-1743, ICD-9-D-1744, ICD-9-D-1745, ICD-9-D-1746, ICD-9-D-1748, ICD-9-D-1749, ICD-9-D-1759, ICD-9-D-2330, ICD-9-D-V103, ICD-9-D-V1389
Ovarian cancer	ICD-10-D-C561, ICD-10-D-C562, ICD-10-D-C569, ICD-10-D-Z8543, ICD-9-D-1830, ICD-9-D-V1043
Uterine cancer	ICD-10-D-C540, ICD-10-D-C541, ICD-10-D-C542, ICD-10-D-C543, ICD-10-D-C548, ICD-10-D-C549, ICD-10-D-C55, ICD-10-D-D070, ICD-10-D-Z8542, ICD-9-D-179, ICD-9-D-1820, ICD-9-D-1821, ICD-9-D-1828, ICD-9-D-2332, ICD-9-D-V1042
Deep vein thrombosis	ICD-10-D-I82401:ICD-10-D-I82409, ICD-9-D-45340:ICD-9-D-45342
Pulmonary embolism	ICD-10-D-I26:ICD-10-D-I269, ICD-9-D-4151:ICD-9-D-4159
Severe liver disease	ICD-10-D-I864, ICD-10-D-K7040, ICD-10-D-K7041, ICD-10-D-K7110, ICD-10-D-K7111, ICD-10-D-K7210, ICD-10-D-K7211, ICD-10-D-K7290, ICD-10-D-K7291, ICD-10-D-K765, ICD-10-D-K766, ICD-10-D-K767, ICD-9-D-4560:ICD-9-D-4562, ICD-9-D-5722:ICD-9-D-5728
Comorbidities	
Obesity	ICD-10-D-E660: ICD-10-D-E669, ICD-9-D-2780, ICD-9-D-27800, ICD-9-D-27801, ICD-9-D-27802, ICD-9-D-27803
Cardiac disorders	ICD-10-D-I441:ICD-10-D-I4435, ICD-10-D-I456, ICD-10-D-I459, ICD-10-D-I470:ICD-10-D-I499, ICD-10-D-R000, ICD-10-D-R001, ICD-10-D-R008, ICD-10-D-T82100:ICD-10-D-T82199S, ICD-10-D-Z4501:ICD-10-D-Z4509, ICD-10-D-Z950, ICD-9-D-4260, ICD-9-D-42610, ICD-9-D-42612, ICD-9-D-42613, ICD-9-D-4267, ICD-9-D-4269, ICD-9-D-4270:ICD-9-D-4274, ICD-9-D-4276:ICD-9-D-4279, ICD-9-D-7850, ICD-9-D-99601, ICD-9-D-99604, ICD-9-D-V450, ICD-9-D-V533, ICD-10-D-I470, ICD-10-D-I471, ICD-10-D-I472, ICD-10-D-I479, ICD-10-D-I480, ICD-10-D-I481, ICD-10-D-I482, ICD-10-D-I483, ICD-10-D-I484, ICD-10-D-I4891, ICD-10-D-I4892, ICD-10-D-I491, ICD-10-D-I492, ICD-10-D-I493, ICD-10-D-I4940, ICD-10-D-I4949, ICD-10-D-I495, ICD-10-D-I498, ICD-10-D-I499, ICD-10-D-R000, ICD-10-D-R001, ICD-10-D-R002, ICD-9-D-4270, ICD-9-D-4271, ICD-9-D-4272, ICD-9-D-42731, ICD-9-D-42732, ICD-9-D-42760, ICD-9-D-42761, ICD-9-D-42769, ICD-9-D-42781, ICD-9-D-42789, ICD-9-D-4279, ICD-9-D-7850, ICD-9-D-7851, ICD-10-D-I0981, ICD-10-D-I501, ICD-10-D-I5020, ICD-10-D-I5021, ICD-10-D-I5022, ICD-10-D-I5023, ICD-10-D-I5030, ICD-10-D-I5031, ICD-10-D-I5032, ICD-10-D-I5033, ICD-10-D-I5040, ICD-10-D-I5041, ICD-10-D-I5042, ICD-10-D-I5043, ICD-10-D-I50810, ICD-10-D-I50811, ICD-10-D-I50812, ICD-10-D-I50813, ICD-10-D-I50814, ICD-10-D-I5082, ICD-10-D-I5083, ICD-10-D-I5084, ICD-10-D-I5089, ICD-10-D-I509, ICD-9-D-39891, ICD-9-D-4280, ICD-9-D-4281
Hypertension	ICD-10-D-I10, ICD-9-D-4010:ICD-9-D-4019
Thyroid disorders	ICD-10-D-E000, ICD-10-D-E001, ICD-10-D-E002, ICD-10-D-E009, ICD-10-D-E010, ICD-10-D-E011, ICD-10-D-E012, ICD-10-D-E018, ICD-10-D-E02, ICD-10-D-E030, ICD-10-D-E031, ICD-10-D-E032, ICD-10-D-E033, ICD-10-D-E034, ICD-10-D-E035, ICD-10-D-E038, ICD-10-D-E039, ICD-10-D-E040, ICD-10-D-E041, ICD-10-D-E042, ICD-10-D-E048, ICD-10-D-E049, ICD-10-D-E0500, ICD-10-D-E0501, ICD-10-D-E0510, ICD-10-D-E0511, ICD-10-D-E0520, ICD-10-D-E0521, ICD-10-D-E0530, ICD-10-D-E0531, ICD-10-D-E0540, ICD-10-D-E0541, ICD-10-D-E0580, ICD-10-D-E0581, ICD-10-D-E0590, ICD-10-D-E0591, ICD-10-D-E060, ICD-10-D-E061, ICD-10-D-E062, ICD-10-D-E063, ICD-10-D-E064, ICD-10-D-E065, ICD-10-D-E069, ICD-10-D-E070, ICD-10-D-E071, ICD-10-D-E0781, ICD-10-D-E0789, ICD-10-D-E079, ICD-9-D-2400, ICD-9-D-2409, ICD-9-D-2410, ICD-9-D-2411, ICD-9-D-2419, ICD-9-D-24200, ICD-9-D-24201, ICD-9-D-24210, ICD-9-D-24211, ICD-9-D-24220, ICD-9-D-24221, ICD-9-D-24240, ICD-9-D-24241, ICD-9-D-24280, ICD-9-D-24281, ICD-9-D-24290, ICD-9-D-24291, ICD-9-D-243, ICD-9-D-2442, ICD-9-D-2448, ICD-9-D-2449, ICD-9-D-2450, ICD-9-D-2451, ICD-9-D-2452, ICD-9-D-2453, ICD-9-D-2454, ICD-9-D-2458, ICD-9-D-2459, ICD-9-D-2460, ICD-9-D-2461, ICD-9-D-2463, ICD-9-D-2468, ICD-9-D-2469, ICD-9-D-78001, ICD-9-D-79094
Depression/Anxiety	Depression: ICD-10-D-F3130,ICD-10-D-F3131,ICD-10-D-F3132,ICD-10-D-F314,ICD-10-D-F315,ICD-10-D-F3160,ICD-10-D-F3161,ICD-10-D-F3162,ICD-10-D-F3163,ICD-10-D-F3164,ICD-10-D-F319,ICD-10-D-F320,ICD-10-D-F321,ICD-10-D-F322,ICD-10-D-F323,ICD-10-D-F324,ICD-10-D-F325,ICD-10-D-F328,ICD-10-D-F329,ICD-10-D-F329,ICD-10-D-F330,ICD-10-D-F331,ICD-10-D-F332,ICD-10-D-F333,ICD-10-D-F3341,ICD-10-D-F3342,ICD-10-D-F338,ICD-10-D-F339,ICD-10-D-F341,ICD-10-D-F348,ICD-10-D-F349,ICD-10-D-F380,ICD-10-D-F381,ICD-10-D-F388,ICD-10-D-F39,ICD-10-D-F412,ICD-10-D-F432,ICD-9-D-2962,ICD-9-D-29621,ICD-9-D-29622,ICD-9-D-29623,ICD-9-D-29624,ICD-9-D-29625,ICD-9-D-29626,ICD-9-D-2963,ICD-9-D-29631,ICD-9-D-29632,ICD-9-D-29633,ICD-9-D-29634,ICD-9-D-29635,ICD-9-D-29636,ICD-9-D-29651,ICD-9-D-29652,ICD-9-D-29653,ICD-9-D-29654,ICD-9-D-29682,ICD-9-D-29682,ICD-9-D-2969,ICD-9-D-29699,ICD-9-D-298,ICD-9-D-3004,ICD-9-D-3090,ICD-9-D-3091,ICD-9-D-30928,ICD-9-D-311 Anxiety Disorders: ICD-10-D-F064,ICD-10-D-F4000,ICD-10-D-F4001,ICD-10-D-F4002,ICD-10-D-F4010,ICD-10-D-F4011,ICD-10-D-F40210,ICD-10-D-F40218,ICD-10-D-F40220,ICD-10-D-F40228,ICD-10-D-F40230,ICD-10-D-F40231,ICD-10-D-F40232,ICD-10-D-F40233,ICD-10-D-F40240,ICD-10-D-F40241,ICD-10-D-F40242,ICD-10-D-F40243,ICD-10-D-F40248,ICD-10-D-F40290,ICD-10-D-F40291,ICD-10-D-F40298,ICD-10-D-F408,ICD-10-D-F409,ICD-10-D-F410,ICD-10-D-F411,ICD-10-D-F413,ICD-10-D-F418,ICD-10-D-F419,ICD-10-D-F42,ICD-10-D-F422,ICD-10-D-F423,ICD-10-D-F424,ICD-10-D-F428,ICD-10-D-F429,ICD-10-D-F430,ICD-10-D-F4310,ICD-10-D-F4311,ICD-10-D-F4312,ICD-10-D-F488,ICD-10-D-F489,ICD-10-D-R452,ICD-10-D-R453,ICD-10-D-R454,ICD-10-D-R455,ICD-10-D-R456,ICD-10-D-R457,ICD-10-D-R4581,ICD-10-D-R4582,ICD-10-D-R4583,ICD-10-D-R4584,ICD-9-D-29384,ICD-9-D-30000,ICD-9-D-30001,ICD-9-D-30002,ICD-9-D-30009,ICD-9-D-30020,ICD-9-D-30021,ICD-9-D-30022,ICD-9-D-30023,ICD-9-D-30029,ICD-9-D-3003,ICD-9-D-3005,ICD-9-D-3009,ICD-9-D-3089,ICD-9-D-30981,ICD-9-D-78095,ICD-9-D-79922,ICD-9-D-79925
Tobacco use	ICD-10-D-F17200,ICD-10-D-F17201,ICD-10-D-F17203,ICD-10-D-F17208,ICD-10-D-F17209,ICD-10-D-F17210,ICD-10-D-F17211,ICD-10-D-F17213,ICD-10-D-F17218,ICD-10-D-F17219,ICD-10-D-F17220,ICD-10-D-F17220,ICD-10-D-F17221,ICD-10-D-F17221,ICD-10-D-F17223,ICD-10-D-F17223,ICD-10-D-F17228,ICD-10-D-F17228,ICD-10-D-F17229,ICD-10-D-F17229,ICD-10-D-F17290,ICD-10-D-F17290,ICD-10-D-F17291,ICD-10-D-F17291,ICD-10-D-F17293,ICD-10-D-F17293,ICD-10-D-F17298,ICD-10-D-F17298,ICD-10-D-F17299,ICD-10-D-F17299,ICD-10-D-Z716,ICD-10-D-Z720,ICD-10-D-Z720,ICD-10-D-Z87891,ICD-9-D-3051,ICD-9-D-98984,ICD-9-D-V1582
Alcohol disorders	ICD-10-D-F1010, ICD-10-D-F1011, ICD-10-D-F10120, ICD-10-D-F10121, ICD-10-D-F10129, ICD-10-D-F1014, ICD-10-D-F10150, ICD-10-D-F10159, ICD-10-D-F10180, ICD-10-D-F10188, ICD-10-D-F1019, ICD-10-D-F1020, ICD-10-D-F1021, ICD-10-D-F10220, ICD-10-D-F10221, ICD-10-D-F10229, ICD-10-D-F10230, ICD-10-D-F10231, ICD-10-D-F10232, ICD-10-D-F10239, ICD-10-D-F1024, ICD-10-D-F10251, ICD-10-D-F10259, ICD-10-D-F1027, ICD-10-D-F10282, ICD-10-D-F10288, ICD-10-D-F1029, ICD-10-D-F10920, ICD-10-D-F10921, ICD-10-D-F10929, ICD-10-D-F10959, ICD-10-D-F10980, ICD-10-D-F1099, ICD-10-D-F3130, ICD-10-D-F3131, ICD-10-D-F3132, ICD-10-D-F314, ICD-10-D-F319, ICD-10-D-F320, ICD-10-D-F321, ICD-10-D-F322, ICD-10-D-F323, ICD-10-D-F329, ICD-10-D-F330, ICD-10-D-F331, ICD-10-D-F332, ICD-10-D-F333, ICD-10-D-F3341, ICD-10-D-F339, ICD-10-D-F341, ICD-10-D-F39, ICD-10-D-F4010, ICD-10-D-F410, ICD-10-D-F411, ICD-10-D-F418, ICD-10-D-F419, ICD-10-D-F430, ICD-10-D-F4310, ICD-10-D-F4312, ICD-10-D-G621, ICD-10-D-I426, ICD-10-D-K2920, ICD-10-D-K2921, ICD-10-D-K700, ICD-10-D-K7010, ICD-10-D-K7011, ICD-10-D-K7030, ICD-10-D-K7031, ICD-10-D-K7040, ICD-10-D-K709, ICD-10-D-M23611, ICD-10-D-M23612, ICD-10-D-O99311, ICD-10-D-O99314, ICD-10-D-Q860, ICD-10-D-S83501A, ICD-10-D-S83502A, ICD-10-D-S83511A, ICD-10-D-S83511D, ICD-10-D-S83511S, ICD-10-D-S83512A, ICD-10-D-S83512D, ICD-10-D-S83512S, ICD-9-D-2910, ICD-9-D-2914, ICD-9-D-29181, ICD-9-D-30300, ICD-9-D-30390, ICD-9-D-30500, ICD-9-D-4255, ICD-9-D-53530, ICD-9-D-5710, ICD-9-D-64844, ICD-9-D-76071
Diabetes	ICD-10-D-E080: ICD-10-D-E139, ICD-9-D-24900: ICD-9-D-25099, ICD-9-D-7915, ICD-9-D-7916
Osteoporosis	ICD-10-D-M810, ICD-10-D-M816, ICD-10-D-M818, ICD-9-D-73300, ICD-9-D-73302, ICD-9-D-73309
Rheumatologic disease	ICD-9-D-4465, ICD-9-D-7100, ICD-9-D-7101, ICD-9-D-7102, ICD-9-D-7103, ICD-9-D-7140, ICD-9-D-7141, ICD-9-D-7142, ICD-9-D-71481, ICD-9-D-71489, ICD-9-D-725, ICD-10-D-M05, ICD-10-D-M06, ICD-10-D-M315, ICD-10-D-M320, ICD-10-D-M3210, ICD-10-D-M3211, ICD-10-D-M3212, ICD-10-D-M3213, ICD-10-D-M3214, ICD-10-D-M3215, ICD-10-D-M3219, ICD-10-D-M328, ICD-10-D-M329, ICD-10-D-M340, ICD-10-D-M341, ICD-10-D-M342, ICD-10-D-M3481, ICD-10-D-M3482, ICD-10-D-M3483, ICD-10-D-M3489, ICD-10-D-M349, ICD-10-D-M353, ICD-10-D-M360
Preoperative steroid injections	ICD-9-D-71511, ICD-9-D-71521, ICD-9-D-71531, ICD-9-D-71591, ICD-9-D-71611, ICD-9-D-71691, ICD-9-D-71801, ICD-9-D-71811, ICD-9-D-71841, ICD-9-D-71851, ICD-9-D-71881, ICD-9-D-71891, ICD-9-D-72610, ICD-9-D-72619, ICD-9-D-8403, ICD-9-D-8404, ICD-9-D-8405, ICD-9-D-72761, ICD-9-D-73341, ICD-10-D-M12511, ICD-10-D-M12512, ICD-10-D-M12519, ICD-10-D-M12811, ICD-10-D-M12812, ICD-10-D-M12819, ICD-10-D-M19011, ICD-10-D-M19012, ICD-10-D-M19019, ICD-10-D-M19111, ICD-10-D-M19112, ICD-10-D-M19119, ICD-10-D-M19211, ICD-10-D-M19212, ICD-10-D-M19219, ICD-10-D-M75100, ICD-10-D-M75101, ICD-10-D-M75102, ICD-10-D-M87011, ICD-10-D-M87012, ICD-10-D-M87019, ICD-10-D-M87111, ICD-10-D-M87112, ICD-10-D-M87119, ICD-10-D-M87211, ICD-10-D-M87212, ICD-10-D-M87219, ICD-10-D-M87311, ICD-10-D-M87312, ICD-10-D-M87319, ICD-10-D-M87811, ICD-10-D-M87812, ICD-10-D-M87819, CPT-20610, CPT-20611
Outcomes	
Infection	ICD-10-D-T8140XA, ICD-10-D-T8140XD, ICD-10-D-T8140XS, ICD-10-D-T8141XA, ICD-10-D-T8141XD, ICD-10-D-T8141XS, ICD-10-D-T8142XA, ICD-10-D-T8142XD, ICD-10-D-T8142XS, ICD-10-D-T8143XA, ICD-10-D-T8143XD, ICD-10-D-T8143XS, ICD-10-D-T8149XA, ICD-10-D-T8149XD, ICD-10-D-T8149XS, ICD-10-D-T814XXA, ICD-10-D-T814XXD, ICD-10-D-T814XXS, ICD-9-D-99851, ICD-9-D-99859
Opioid use	USC-02211, USC-02212, USC-02214, USC-02221, USC-02222, USC-02232
TSA	CPT-23472, ICD-10-P-0RRJ00Z, ICD-10-P-0RRJ07Z, ICD-10-P-0RRJ0J6, ICD-10-P-0RRJ0J7, ICD-10-P-0RRJ0JZ, ICD-10-P-0RRJ0KZ, ICD-10-P-0RRK00Z, ICD-10-P-0RRK07Z, ICD-10-P-0RRK0J6, ICD-10-P-0RRK0J7, ICD-10-P-0RRK0JZ, ICD-10-P-0RRK0KZ, ICD-9-P-8188, ICD-9-P-8180
Frozen shoulder	ICD-10-D-M7500, ICD-10-D-M7501, ICD-10-D-M7502
Stroke	ICD-9-D-4151, ICD-10-D-I6300, ICD-10-D-I63011, ICD-10-D-I63012, ICD-10-D-I63013, ICD-10-D-I63019, ICD-10-D-I6302, ICD-10-D-I63031, ICD-10-D-I63032, ICD-10-D-I63033, ICD-10-D-I63039, ICD-10-D-I6309, ICD-10-D-I6310, ICD-10-D-I63111, ICD-10-D-I63112, ICD-10-D-I63113, ICD-10-D-I63119, ICD-10-D-I6312, ICD-10-D-I63131, ICD-10-D-I63132, ICD-10-D-I63133, ICD-10-D-I63139, ICD-10-D-I6319, ICD-10-D-I6320, ICD-10-D-I63211, ICD-10-D-I63212, ICD-10-D-I63213, ICD-10-D-I63219, ICD-10-D-I6322, ICD-10-D-I63231, ICD-10-D-I63232, ICD-10-D-I63233, ICD-10-D-I63239, ICD-10-D-I6329, ICD-10-D-I6330, ICD-10-D-I63311, ICD-10-D-I63312, ICD-10-D-I63313, ICD-10-D-I63319, ICD-10-D-I63321, ICD-10-D-I63322, ICD-10-D-I63323, ICD-10-D-I63329, ICD-10-D-I63331, ICD-10-D-I63332, ICD-10-D-I63333, ICD-10-D-I63339, ICD-10-D-I63341, ICD-10-D-I63342, ICD-10-D-I63343, ICD-10-D-I63349, ICD-10-D-I6339, ICD-10-D-I6340, ICD-10-D-I63411, ICD-10-D-I63412, ICD-10-D-I63413, ICD-10-D-I63419, ICD-10-D-I63421, ICD-10-D-I63422, ICD-10-D-I63423, ICD-10-D-I63429, ICD-10-D-I63431, ICD-10-D-I63432, ICD-10-D-I63433, ICD-10-D-I63439, ICD-10-D-I63441, ICD-10-D-I63442, ICD-10-D-I63443, ICD-10-D-I63449, ICD-10-D-I6349, ICD-10-D-I6350, ICD-10-D-I63511, ICD-10-D-I63512, ICD-10-D-I63513, ICD-10-D-I63519, ICD-10-D-I63521, ICD-10-D-I63522, ICD-10-D-I63523, ICD-10-D-I63529, ICD-10-D-I63531, ICD-10-D-I63532, ICD-10-D-I63533, ICD-10-D-I63539, ICD-10-D-I63541, ICD-10-D-I63542, ICD-10-D-I63543, ICD-10-D-I63549, ICD-10-D-I6359, ICD-10-D-I636, ICD-10-D-I638, ICD-10-D-I6381, ICD-10-D-I6389, ICD-10-D-I639
Acute myocardial infarction	ICD-10-D-I2101, ICD-10-D-I2102, ICD-10-D-I2109, ICD-10-D-I2111, ICD-10-D-I2119, ICD-10-D-I2121, ICD-10-D-I2129, ICD-10-D-I213, ICD-10-D-I214, ICD-10-D-I219, ICD-10-D-I21A1, ICD-10-D-I21A9, ICD-10-D-I220, ICD-10-D-I221, ICD-10-D-I222, ICD-10-D-I228, ICD-10-D-I229, ICD-9-D-41001, ICD-9-D-41011, ICD-9-D-41021, ICD-9-D-41031, ICD-9-D-41051, ICD-9-D-41071, ICD-9-D-41081, ICD-9-D-41091
Pulmonary embolism	ICD-10-D-I26:ICD-10-D-I269, ICD-9-D-4151:ICD-9-D-4159
Deep vein thrombosis	ICD-10-D-I82401:ICD-10-D-I82409, ICD-9-D-45340:ICD-9-D-45342
